# Elimination of classically-activated macrophages in tumor-conditioned medium by alternatively-activated macrophages

**DOI:** 10.1242/bio.027300

**Published:** 2017-11-21

**Authors:** Fidel-Nicolás Lolo, Cristina Rius, Sergio Casas-Tintó

**Affiliations:** 1 Molecular Oncology Programme, Spanish National Cancer Centre (CNIO), Melchor Fernández Almagro, 3, Madrid 28034, Spain; 2 Cell and Developmental Biology Area, Centro Nacional de Investigaciones Cardiovasculares Carlos III (CNIC), Melchor Fernández Almagro, 3, Madrid 28029, Spain; 3 Laboratory of Molecular and Genetic Cardiovascular Pathophysiology, Centro Nacional de Investigaciones Cardiovasculares (CNIC) and CIBER de Enfermedades Cardiovasculares, Melchor Fernández Almagro, 3, 28029 Madrid, Spain; 4 Department of Pharmacy and Biotechnology, School of Biomedical and Health Sciences, Universidad Europea de Madrid, C/ Tajo s/n, Villaviciosa de Odón, Madrid 28670, Spain; 5 Molecular, Cellular and Developmental Neurobiology Department, Cajal Institute (CSIC), Avda. Doctor Arce, 37, Madrid 28002, Spain

**Keywords:** Classically-activated macrophages, Alternatively-activated macrophages, Tumoral environment, Apoptosis

## Abstract

Cellular interactions are critical during development, tissue fitness and epithelial tumor development. The expression levels of specific genes confer to tumoral cells a survival advantage versus the normal neighboring cells. As a consequence, cells surrounding tumors are eliminated and engulfed by macrophages. We propose a novel scenario in which circulating cells facing a tumor can reproduce these cellular interactions. *In vitro* cultured macrophages from murine bone marrow were used to investigate this hypothesis. M1 macrophages in tumoral medium upregulated markers of a suboptimal condition, such as *Sparc* and *TyrRS*, and undergo apoptosis. However, M2 macrophages display higher *Myc* expression levels and proliferate at the expense of M1. Resulting M1 apoptotic debris is engulfed by M2 in a Sparc- and TyrRS-dependent manner. These findings suggest that tumor-dependent macrophage elimination could deplete immune response against tumors. This possibility could be relevant for macrophage based anti-tumoral strategies.

## Abstract (precis)

**Summary:** Cell-autonomous death and engulfment could play an important role in regulating tumor progression and should be taken into account when considering the behavior of pro- and anti-tumoral cells in the complex tumor environment. This article has an associated First Person interview with the first author of the paper as part of the supplementary information.

## INTRODUCTION

Therapeutic strategies in oncology include the adoptive transfer of anti-tumoral classically-activated macrophages (CAMs, also referred to as M1) (Eymard et al., 1996). *Ex vivo* programmed CAMs have the potential to induce regression of established tumors (Shiao et al., 2011; Andreesen et al., 1990). However, transfer of CAMs for cellular therapy has not reached the expected results so far; exogenous activated macrophages show restricted motility and become rapidly undetectable when facing the tumor microenvironment (Shiao et al., 2011; Andreesen et al., 1990; Tveita et al., 2014). We rationalized that transferred exogenous CAMs used in cellular anti-tumoral therapy might be somehow eliminated, thus compromising the efficiency of treatment. To study that, we use an *in vitro* model of polarized macrophages, M1, which are key effector cells for the elimination of cancer cells, and M2, which promote tumoral growth (Italiani and Boraschi, 2014), to study their behavior under tumoral conditions. Our results show that M1 upregulated Secreted Protein, Acidic, Cysteine-Rich (SPARC) and Tyrosyl-tRNA synthetase (TyrRS), which have previously been shown as markers of compromised cellular fitness (Casas-Tintó et al., 2015; Portela et al., 2010). Concomitantly, M1 macrophages undergo apoptosis and are finally engulfed by M2 macrophages. Based on these observations, we propose that adoptive transfer of macrophages as an anti-tumor therapy might undergo CAM elimination, and can have an impact on the effectiveness of the treatment.

## RESULTS

**Fig. 1.**
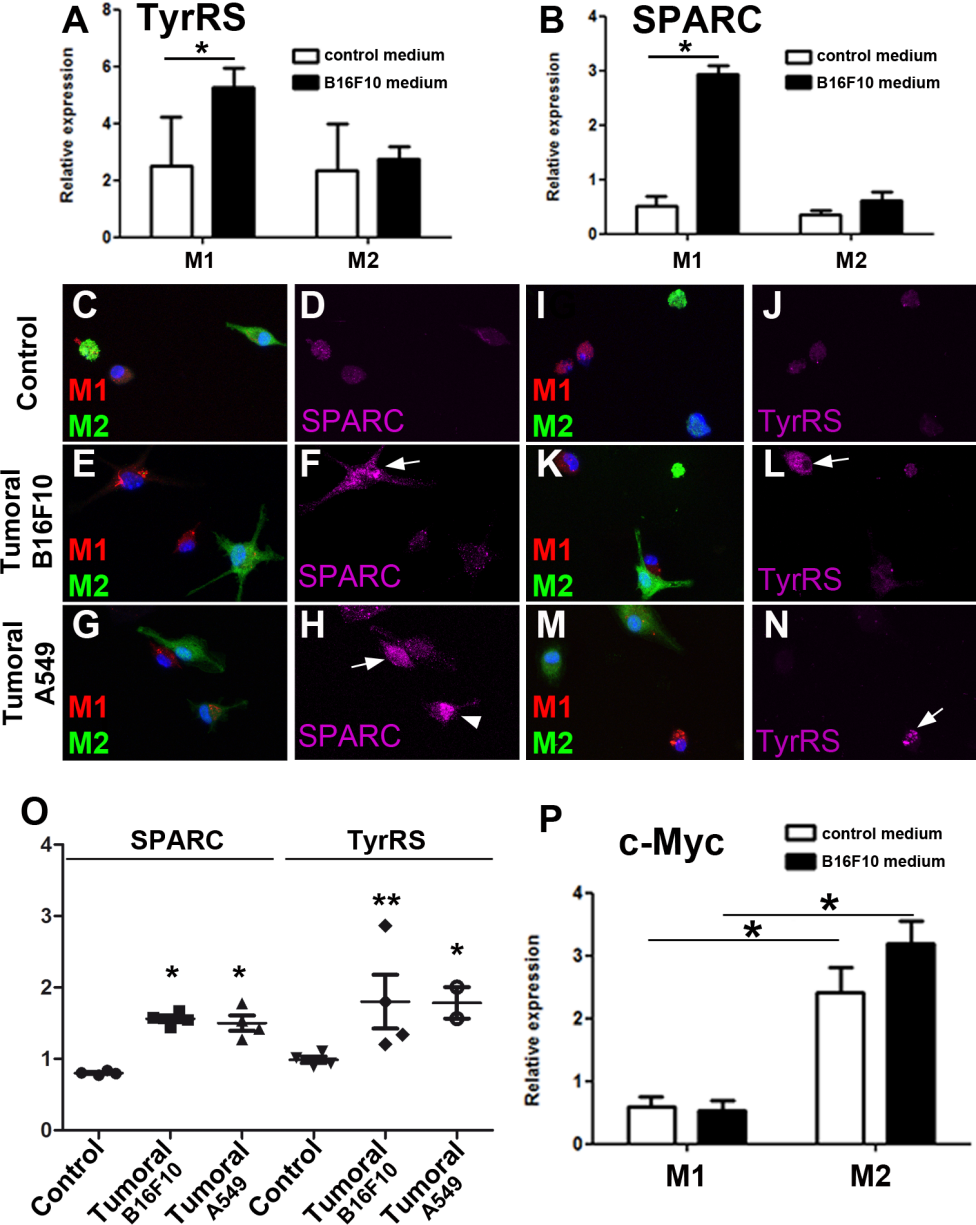
**M1 and M2 macrophage characterization** **in tumoral-conditioned medium.** (A,B) qRT-PCR relative expression levels of *TyrRS* and *SPARC* in M1 and M2 macrophages cultured in control medium (white bars) or B16F10-derived tumoral medium (black bars) for 24 h. The analysis was performed twice, with three replicates each time. Data are mean±s.e.m. Statistical significance was calculated using a *t*-test, with significant differences between compared groups noted by *P<0.05. (C-N) Immunostaining against TyrRS or SPARC (both shown in magenta) of M1 (red) and M2 (green) macrophages cultured in control medium (C,D,I,J) or in two different types of tumor-conditioned medium: B16F10-derived tumoral medium (E,F,K,L) or A549-derived tumoral medium (G,H,M,N) for 24 h. DAPI is shown in blue. Arrows indicate cells positive for the specific staining. (O) Quantification of SPARC and TyrRS mean fluorescent intensity staining under different cultured conditions; 10 to 30 individual cells were analyzed in each case. Statistical significance was calculated using one-way ANOVA Bonferroni's Multiple Comparison Test, with significant differences between compared groups noted by *P<0.05, **P<0.01. (P) Relative expression levels of *c-Myc* in M1 and M2 macrophages cultured in control medium (white bars) or B16F10-derived tumoral medium (black bars) for 24 h.

To characterize whether anti-tumor macrophages are compromised under tumoral conditions, we analyzed SPARC and TyrRS expression. We used an *in vitro* model of murine bone marrow-derived M1 and M2 macrophages. Polarization was validated using specific M1 and M2 markers (Fig. S1) (Quatromoni and Eruslanov, 2012). M1 and M2 were separately cultured either in control, B16F10-derived or A-549-derived tumoral media for 24 h. M1 showed a significant increase of *SPARC* and *TyrRS* expression both at mRNA (Fig. 1A,B) and protein levels when cultured in tumor-conditioned medium, compared to M2 (Fig. 1C-N, M1 protein levels quantified in Fig. 1O).

**Fig. 2.**
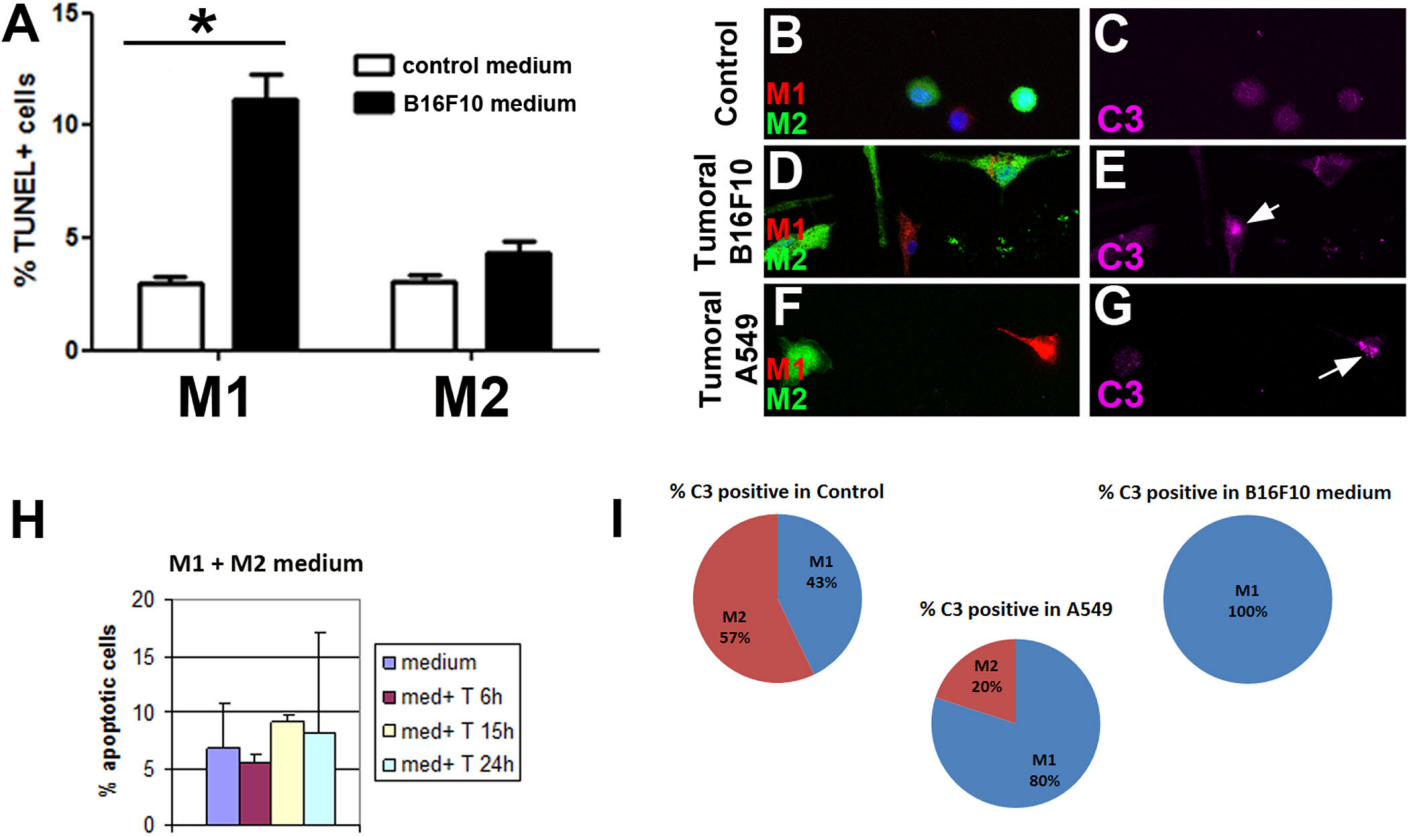
**M1 macrophages undergo apoptosis when cultured in tumoral medium as compared to M2.** (A) Percentage of TUNEL-positive M1 or M2 macrophages cultured in control medium (white bars) or in B16F10-derived tumoral medium (black bars) for 24 h. Data are mean±s.e.m. Statistical significance was calculated using a *t*-test, with significant differences between compared groups noted by *P<0.05. (B-G) Immunostaining against Caspase-3 (magenta) of M1 (red) or M2 (green) macrophages cultured in control medium (B,C) or in two different types of tumor-conditioned medium: B16F10-derived tumoral medium (D,E) or A549-derived tumoral medium (F,G) for 24 h. DAPI is shown in blue. Arrows indicate cells positive for the specific staining. (H) Percentage of annexin V+ apoptotic M1 macrophages after being cultured in M2-derived medium for the indicated time points. Data are mean±s.d. (I) Percentage of Caspase-3 positive M1 or M2 macrophages in control medium, B16F10-derived tumoral medium or A549-derived tumoral medium.

These results suggest that M1 show a compromised fitness compared to M2. In addition M2 cells upregulated c-Myc expression as compared to M1 (Pello, 2016; Pello et al., 2012a,b; Cano-Ramos et al., 2014) (Fig. 1P). In line with these results, slightly increased levels of c-Myc confer advantageous properties to *Drosophila* and mammalian epithelial cells (Moreno and Basler, 2004; Clavería et al., 2013; Morata and Ballesteros-Arias 2015), whereas lower levels of c-Myc determine a suboptimal state (Johnston et al., 1999). Altogether, these data indicate that M1 cells are tagged as suboptimal cells in this tumoral context. To determine whether M1 cells are eliminated in a tumoral medium but M2 survive, we studied apoptosis response after culturing M1 and M2 separately in either control, B16F10-derived or A-549-derived tumoral media for 24 h. There were no differences in TUNEL-positive cells between M1 and M2 cells in control medium (Fig. 2A). However, we observed that only M1 macrophages underwent apoptosis significantly when cultured in tumoral-conditioned medium (Fig. 2A). This observation was further confirmed by active Caspase-3 staining, which showed upregulation specifically in M1 after 24 h of culture in tumor-conditioned media (Fig. 2B-G, quantified in Fig. 2I). Then, to rule out the possible apoptotic effect of M2-secreted factors during this time-period, we cultured M1 in M2-derived medium and analyzed the number of apoptotic cells. There were no significant differences in the percentages of apoptotic M1 and M2 macrophages at the different time-points analyzed (Fig. 2H; FACS in Fig. S2). These results show that M1 autonomously activate apoptosis in response to tumor-conditioned medium.

**Fig. 3.**
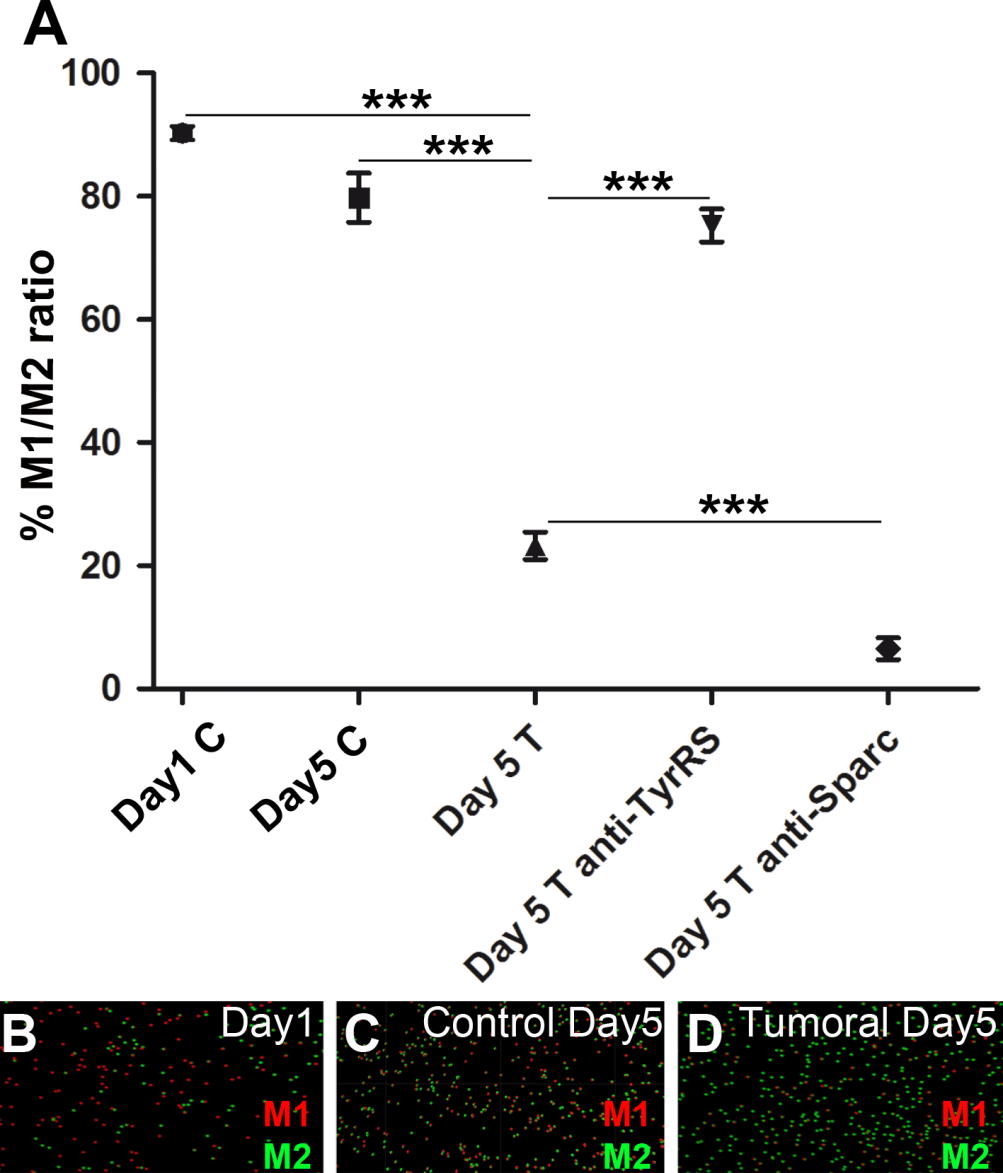
**M1 are engulfed by M2.** (A) Quantification of the ratio of M1/M2 cultured in control medium or B16F10-derived tumoral medium for 1 or 5 days. Antibodies against TyrRS and SPARC were added to B16F10-derived medium for 5 days were indicated. All measurements are relative to M1/M2 ratio cultured in control medium at day 1. Data are mean±s.e.m. Statistical significance was calculated using a one-way ANOVA Bonferroni's Multiple Comparison Test (***P<0.001). (B-D) Representation from Imaris (Bitplane) of M1 (red dots) and M2 (green dots) after 1 day or 5 days of culture in control or B16F10-derived medium. The representation in B corresponds to both control and tumoral medium as there were no differences after 1 day of incubation between these two conditions.

Previous reports have shown that a population of phagocytic cells contribute to the elimination of apoptotic cells (Lolo et al., 2012). We therefore decided to characterize whether M2 could participate in the elimination of anti-tumoral M1 once apoptosis is activated. We co-cultivated M1 and M2 macrophages at a 1:1 proportion (previously labeled with different color-cell trackers, see Materials and Methods) in control or B16F10-derived tumoral medium. After 24 h, 48 h and 5 days of co-culture, we quantified the number of each population; our results show that the ratio M1/M2 remained unaffected when co-cultured in control medium. However, when co-cultured in B16F10-derived tumoral medium the M1 population was reduced compared to M2 (Fig. 3A-D; Table S1, Figs S3 and S4). To evaluate our hypothesis of an active mechanism to eliminate M1, we blocked TyrRS and SPARC signaling, adding specific antibodies to the cell culture medium. TyrRS is secreted by cells to be eliminated and stimulates the recruitment of macrophages that eliminate the apoptotic bodies (Casas-Tintó et al., 2015). When anti-TyrRS was added to the cell culture, M1 elimination was prevented (Fig. 3A; Table S1). This result goes in line with previous reports which postulated the existence of a loser killing signal in a cell competition *in vitro* model (Senoo-Matsuda and Johnston, 2007), and suggests that TyrRS secretion would induce M2 recruitment and therefore might function as a signal to engulf M1 cells. On the other hand, SPARC is a protective signal for suboptimal cells. We incubated the cell culture with anti-SPARC to block SPARC protective function; consequently M1 cells were eliminated more efficiently (Fig. 3A; Table S1). To validate whether M2 cells are engulfing M1-loser cells we performed live imaging of co-cultured cells. The results showed that M2 engulfed M1 when cultured in the B16F10-derived tumoral medium (Movies 1-4). Interestingly, engulfment events were observed as early as 6-7 h of co-culture (Movie 2). According to our previous observations, autonomously tumor-induced M1 apoptosis is not yet induced at this time point. These data suggest that engulfment could be the cause of M1 elimination in the presence of M2. A similar conclusion was previously raised in *Drosophila*, where engulfment genes were shown to be required for apoptosis (Li and Baker, 2007). Our results might therefore indicate that M1 behave as suboptimal cells in response to tumor signals, activate apoptosis and then are engulfed by M2, which are the tumor-associated macrophages. To discriminate as to whether engulfment is the cause or rather the consequence of apoptosis in the context of a complex tumor microenvironment remains to be elucidated in the future.

**Fig. 4.**
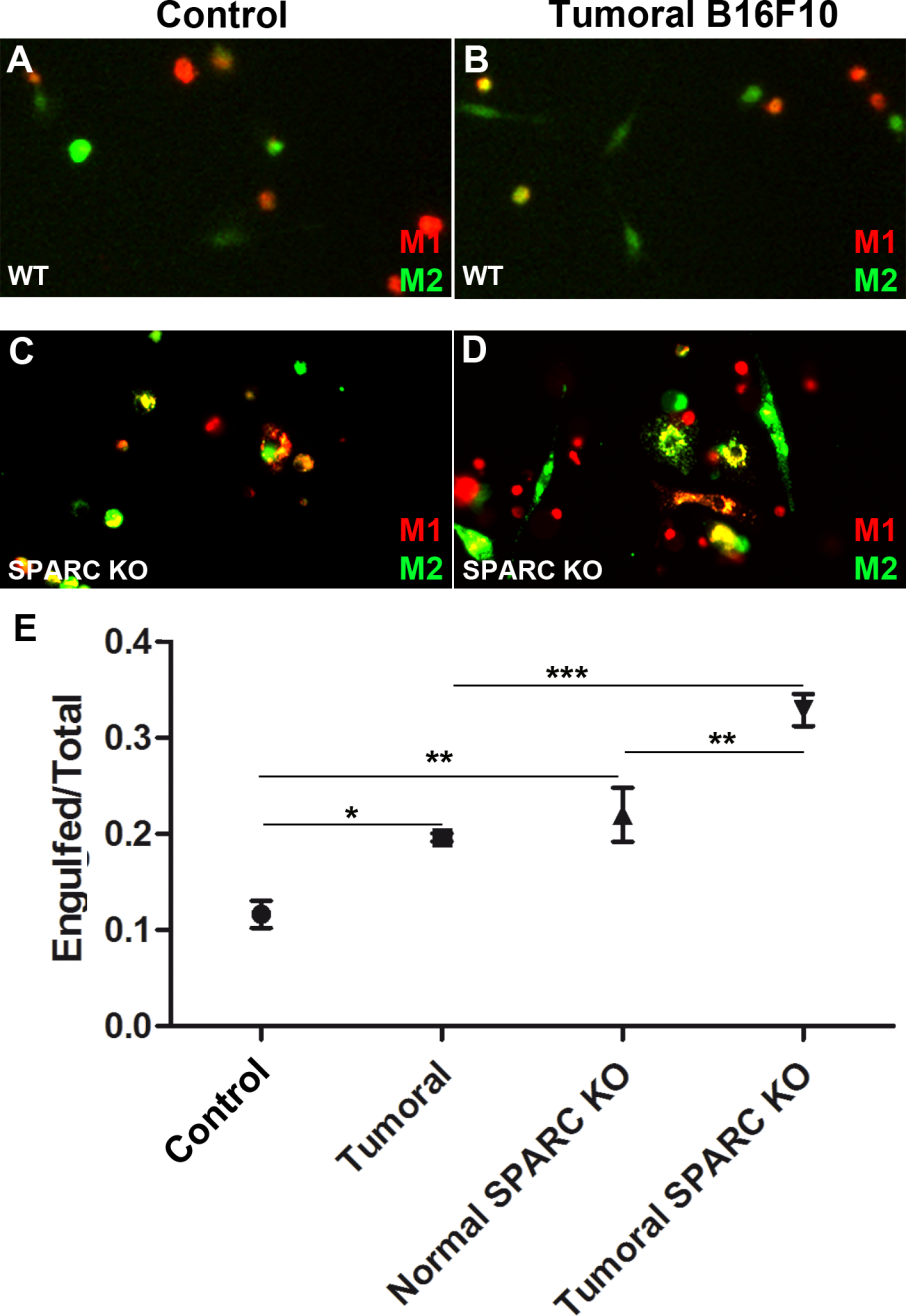
**SPARC-depleted M1 macrophages are more efficiently engulfed by M2 macrophages under tumoral conditions.** (A-D) Wild-type or SPARC-depleted M1 (red) and M2 (green) macrophages cultured in control medium (A,C) or B16F10-derived tumoral medium (B,D). (E) Quantification of the number of engulfed M1 macrophages over the total in control or B16F10-derived tumoral medium comparing co-culture of wild-type macrophages (WT) with SPARC KO ones. Data are mean±s.e.m. Statistical significance was calculated using a one-way ANOVA Bonferroni's Multiple Comparison Test (*P<0.05, **P<0.01, ***P<0.001).

Finally, to validate that SPARC works as a protective signal for anti-tumoral macrophages, we tested M1 elimination using bone-marrow derived M1 and M2 from an SPARC KO mouse (Fig. 4A-E). M1 cells were unable to upregulate SPARC, and therefore do not activate the protective signaling dependent on SPARC signaling. Under these conditions, the number of phagocytic events (fragments of M1 inside M2) was significantly higher and M1 macrophages were eliminated more efficiently as compared to a wild-type background (Fig. 4A-D, quantification in Fig. 4E; Table S2). Consistently, a role for SPARC has been previously associated to an increase in M2 versus M1 ratio in a murine pancreatic cancer model, suggesting that M1 could also be eliminated *in vivo* (Arnold et al., 2012).

## DISCUSSION

Although we cannot rule out that the decreased number of injected CAMs *in vivo* could be partially due to a reprogramming of these CAMs into other macrophage types or rather tumor medium-induced apoptosis, our results indicate that anti-tumoral macrophages can also be engulfed by pro-tumoral associated macrophages, as we have observed in our *in vitro* model. This possibility could explain why CAM-based anti-tumoral therapy has not reached the expected efficiency. Expression of specific markers and regulators of compromised cellular fitness, such as SPARC and TyrRS, identify M1 as suboptimal cells. Consequently, these cells are eliminated and reduce their anti-tumoral effectiveness. Although still preliminary, these observations might lead to the future prospect of decreasing CAMs suboptimal behavior in a way that their lifetime in the organism would be lengthened, increasing their efficiency in fighting the tumor. Future work with *in vivo* experiments of adoptively transferred macrophages would be required to address this possibility.

TyrRS has a dual function; under control conditions it is a Tyrosyl-transferase essential for protein synthesis. However, under certain situations in which the cell integrity is compromised, TyrRS is upregulated, secreted and cleaved to recruit phagocytic cells (Casas-Tintó et al., 2015). Because of these two independent functions, we believe that TyrRS is not an ideal therapeutic target. Although it might be interesting to evaluate the effects of blocking TyrRS in the tumor microenvironment, the side effect on healthy cells make this strategy less attractive. However, SPARC has been shown to protect cells from apoptosis *in vitro* via activation of integrin-linked kinase and AKT (Weaver et al., 2008) and prevents the elimination of suboptimal cells (Portela et al., 2010). We have shown here that initial expression of SPARC in M1 is probably a protective signal and only after a continuous exposure to the apoptotic signal, SPARC endogenous expression is overwhelmed and not enough to impede M1 elimination *in vitro*. According to these observations, we consider that specifically modulation of SPARC levels could be an interesting strategy to increase/improve CAMs survival rate. Following studies will be aimed to investigate if SPARC expression in CAMs could be upregulated by modulating ligand-mediated intracellular pathways like TGF-β (Shibata and Ishiyama, 2013), c-Jun (Briggs et al., 2002) and Snail (Grant et al., 2014) activity. Alternatively, overexpression of SPARC by gene-adoptive transfer could be also a suitable approach to reduce looseness of CAMs.

Overall, we propose that cell-autonomous cell death and the concomitant engulfment could play an important role in regulating tumor progression and should be taking into account when considering the behavior of pro- and anti-tumoral cells in the complex tumor environment.

## MATERIAL AND METHODS

### Murine macrophages and culture conditions

#### Mice and care

Wild-type (WT) mice (C57BL/6 background) were purchased from The Jackson Laboratory, Madison, WI. SPARC^−/−^ femurs and tibiae were a gift from Dr P. P. López-Casas at the Spanish National Cancer Centre (CNIO); the corresponding SPARC^−/−^ mice (C57BL/6 background) were sacrificed at CNIO following approved procedures by the CNIO Research Ethics Committee. Mice were maintained on a standard diet (Panlab, Barcelona, Spain). Care of animals was in accordance with institutional guidelines and regulations, and conformed to EU Directive 86/609/EEC and Recommendation 2007/526/EC regarding the protection of animals used for experimental and other scientific purposes, enacted under Spanish law 1201/2005. All animal procedures have been approved by the Spanish National Cardiovascular Centre (CNIC) or CNIO Research Ethics Committees.

#### Isolation of bone marrow cells

Bone marrow (BM)-derived cells were harvested from WT and SPARC^−/−^ mice. Briefly, mice were euthanized by carbon dioxide inhalation. Twelve mice (8 weeks of age) were culled and femur and tibia were rapidly harvested. Skin, skeletal muscle and fat tissue surrounding the bones were removed.

Both bones ends were cut and the BM was flushed with Hank's Balanced Salt Solution (HBSS) containing 2 mM EDTA using a 1-ml insulin syringe with a 27 G needle. The obtained BM was disaggregated by pipetting and washed with PBS. Erythrocytes were lysed using lysis buffer (KH4Cl 0.15 M, KHCO3 0.01 M, EDTA.N2 0.01 M, pH7.4). BM cells were cultured (2×10^6^ cells/ml) for 7 days with DMEM medium supplemented with inactivated FBS 10% and M-CSF (100 ng/ml) to obtain 95%-pure BM-derived non-activated macrophages and finally polarized into M1 or M2 using the following media for 48 h: for M1 phenotype, complete DMEM (with 10% inactivated FBS) plus lipopolysaccharide, LPS (10 ng/ml) and interferon gamma, IFNy (10 ng/ml); for M2 phenotype, complete DMEM (with 10% inactivated FBS) plus interleukin-4, IL-4 (20 ng/ml).

The tumoral medium was obtained as previously described (Weaver et al., 2008). Briefly, B16-F10 murine melanoma cells or A549 adenocarcinoma human alveolar epithelial cells (ATCC^®^) were cultured in DMEM supplemented with 10% FCS, L-glutamine and penicillin/streptomycin. Once grown to 90% confluence, medium was discarded, and flasks were rinsed twice with PBS. Cells were then incubated with fresh complete DMEM for 24 h; the tumor-cell–conditioned medium was collected, filtered (0.20 µm) and stored at −20°C. Control medium was normal DMEM supplemented with 10% FCS, L-glutamine and penicillin/streptomycin or conditioned medium derived from a fibroblast cell line (CCM). Both tumoral and control conditioned media were mixed (3:1) with fresh DMEM to compensate for the possible lack of certain metabolites.

### Quantitative RT-PCR

Total RNA was isolated from M1 and M2 (Trizol, Invitrogen) and cDNAs were synthetized with M-MLV RT (Invitrogen). The following specific primers were used:

c-MYC-Forward: 5′ GAGCTGTTTGAAGGCTGGATTT 3′

c-MYC*-*Reverse: 5′ TCCTGTTGGTGAAGTTCACGTT 3′

SPARC-Forward: 5′ TAAACCCCTCCACATTCCTG 3′

SPARC*-*Reverse: 5′ CACGGTTTCCTCCTCCACTA 3′

TyrRS-Forward: 5′ GCAGGAGGTTCTAGGGGAAG 3′

TyrRS*-*Reverse: 5′ GGCTTTCATGTTGTCCAGGT 3′

Quantitative reverse transcription polymerase chain reaction (qRT-PCR) was performed using SYBR^®^-green (Applied Biosystems) using a 7500 Real Time PCR System (Applied Biosystems) with cycling conditions of 95°C for 10 min and 40 cycles of 95°C for 15 s and 55°C for 1 min. Each experimental point was performed with samples from two mice and three replicates per experimental point, and differences were assessed with a two-tailed Student *t*-test. Results were normalized using the housekeeping GAPDH and the ΔΔ cycle threshold method and are expressed as the relative change (-fold) of the stimulated group over the control group, which was used as a calibrator. qRT-PCR results were analyzed with 7500 v2.0.6 software (Applied Biosystems).

### Immunostaining

Polarized M1 and M2 cells were co-cultured in a 1:1 ratio for 48 h in a 60 mm petri dish. Cells were fixed with 4% formaldehyde in phosphate-buffered saline for 10 min, washed three times with 0.1% triton, incubated with primary antibodies: anti TyrRS (1:100, Abnova, Taipei City, Taiwan, #H00008565-M02) (Niehues et al., 2015), anti SPARC (1:200, Cell Signaling Technology, #5420) (Fukunaga-Kalabis et al., 2008), or anti caspase 3 (1:100, Cell Signaling Technology, #9661, Lim et al., 2017), and secondary antibodies Alexa 647 (Life Technologies) and mounted in Vectashield mounting medium with DAPI. Preparations were imaged by confocal microscopy with a SP5 microscope (Leica, Wetzlar, Germany). Fluorescence quantification and cell counting was performed with Imaris 6.3.1 (Bitplane).

### Apoptosis and cell viability assays

Macrophage apoptosis was measured by an *In situ* Cell Death detection kit in M1 and M2 macrophages (TUNEL staining, Roche) and Phosphatidylserine (PS) externalization in M1 macrophages; briefly, M1 were harvested by trypsinization and washed twice with PBS. Washed cells were resuspended in 200 μl binding buffer (PBS containing 1 mM calcium chloride). FITC-conjugated annexin V (0.5 μg ml^−1^ final concentration) and propidium iodide (PI; 1 μg ml^−1^ final concentration) were added according to the manufacturer's instructions (Biosea, Beijing, China). After incubation for 20 min at room temperature, 400 μl binding buffer was added, and samples were immediately analysed on a FACS Calibur flow cytometer (Becton Dickinson, New Jersey, USA) with excitation using a 488 nm argon ion laser. PI was added to samples to distinguish necrotic or late apoptotic events (annexin V+, PI+) from early apoptotic (annexin V+, PI−) and viable cells (annexin V−, PI−).

### Quantification of M1/M2

CellTracker Red CMTPx (Molecular Probes) and Green CMFDA (Molecular Probes) were used to mark M1 and M2, respectively. Quantification was performed using Imaris (Bitplane) software. All the cells from a six-well plate were counted; each cell was identified as a color-coded dot. The total number of cells was determined counting the red (M1) or green (M2), then the number of red signals within the green cells was measured to establish engulfment events. The ratio M1/M2 was represented as the average of three independent experiments.

### Live videos

Cultured M1 or M2 macrophages were mechanically detached from culture plates with scrapers and collected in 15 ml falcons separately. They were then centrifuged at 1200 rpm for 5 min, the supernatant was discarded and the pellet resuspended in 1 ml RPMI medium without serum. M1 macrophages were labeled with 1 μl CellTracker Red CMTPx and M2 macrophages with 1 μl CellTracker Green CMFDA. After the 30-min incubation, 10 ml complete RPMI medium (with 10% FBS) were added. The cells were centrifuged again at 1200 rpm for 5 min and washed with PBS. After counting on a Neubauer chamber the same number of M1 and M2 macrophages were mixed, plated on eight-well plates (Ibidi) and incubated with control medium (DMEM or CCM) or B16F10-derived tumoral medium. Cells were imaged on a time-lapse microscope every 10 min during 24 h.

### Statistical analysis

Statistical significance was calculated using ANOVA Bonferroni's Multiple Comparison Test (*P<0.05, **P<0.01, ***P<0.001) or *t*-test (*P<0.05).

Mean fluorescent intensity was analyzed with ImageJ (https://imagej.nih.gov/ij/) using CTCF index, which takes into account the integrated density−(area of selected cell×mean fluorescence of background readings). This index corrects for intensity, background and cell size as it is shown elsewhere (McCloy et al., 2014; Burgess et al., 2010).

## Supplementary Material

Supplementary information

First Person interview
