# Supplementary material for: Elimination of classically-activated macrophages in tumor-conditioned medium by alternatively-activated macrophages
Source: Biol Open. 2017 Nov 21;6(12):1897–903. doi: 10.1242/bio.027300 (PMC5769648; doi:10.1242/bio.027300)
Supplement: Supplementary information [file biolopen-6-027300-s1.pdf]

FIRST PERSON

First person – Fidel-Nicolás Lolo Romero

First Person is a series of interviews with the first authors of a selection of papers published in Biology Open, helping early-career researchers promote themselves alongside their papers. Fidel-Nicolás Lolo Romero is first author on 'Elimination of classically-activated macrophages in tumor-conditioned medium by alternatively-activated macrophages', published in BiO. Fidel is a postdoc in the lab of Miguel Ángel del Pozo at the Spanish National Cardiovascular Research Centre, Madrid, Spain, investigating molecular oncology, cell biology, biophysics, *Drosophila* and mouse genetics.

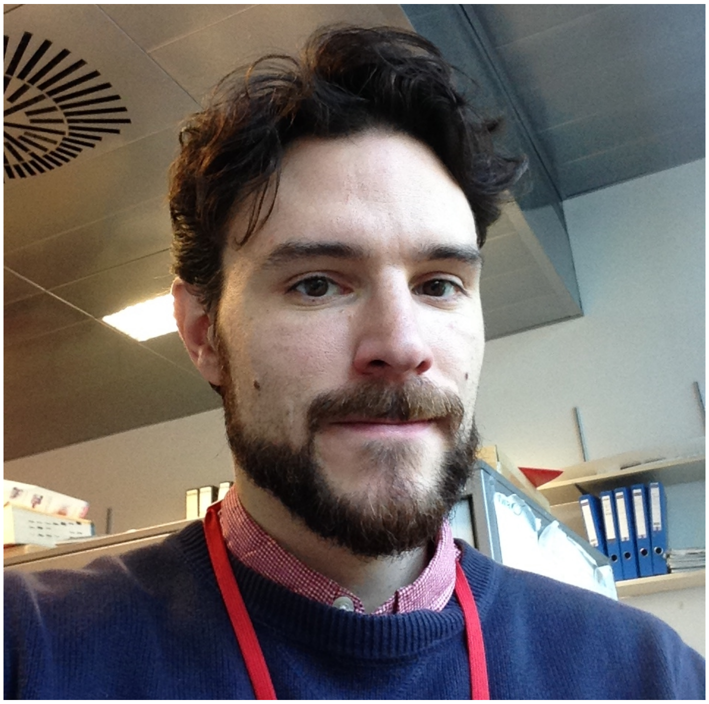

Fidel-Nicolás Lolo Romero

What is your scientific background and the general focus of your lab?

I did my bachelor's degree in biology at Universidad de Alcalá de Henares (Madrid). In 2006, I did a master's on cellular signalling at the same university and then, a PhD in molecular oncology at CNIO (Spanish National Cancer Research Centre). During the thesis period, I studied cell competition, the process of selection among cells of the same organism by means of cellular fitness, using *Drosophila melanogaster* as a model organism; the work published in this paper relates to these previous studies. During my PhD, I had the opportunity to learn many different molecular biology techniques as well as fly genetics to obtain transgenic animals. The work done during those years rendered a number of publications where we were able to show some of the molecular details behind the process of cell competition. After that, I moved to CNIC (Spanish National Cardiovascular Research Centre) where I have worked since 2012. My present project aims to understand the molecular mechanisms that underpin mechanosensing – the ability of cells to sense mechanical forces. From the very beginning of my postdoctoral training, I started a collaboration with biophysicists to study this process in depth. The results of these investigations might shed light on diseases such as cancer and atherosclerosis.

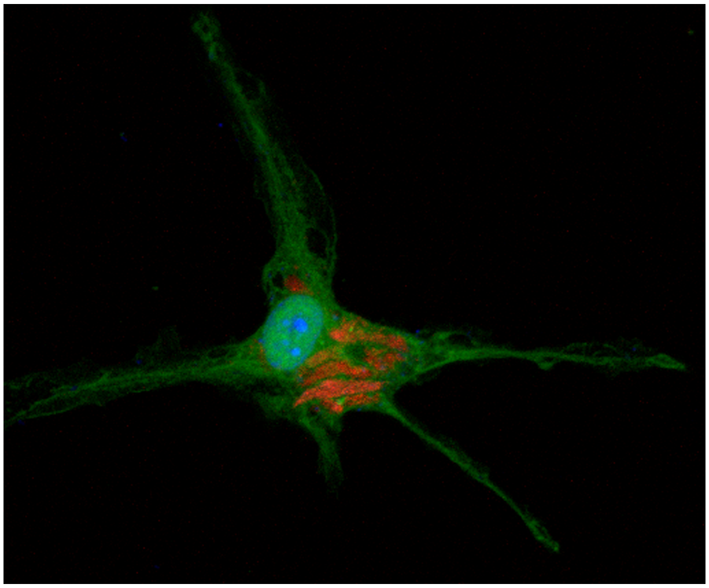

Confocal microscopy of an M2 bone marrow-derived macrophage (green), cultured in a tumour-conditioned medium for 48 hours. An M1 bone marrow-derived macrophage appears as remaining leftovers (red) after being engulfed the M2 macrophage. DAPI is shown in blue.

Fidel-Nicolás Lolo Romero's contact details: Mechanoadaptation and Caveolae Biology Group, Spanish National Cardiovascular Research Centre, Madrid 28029, Spain.

E-mail: flolo@cnic.es

How would you explain the main findings of your paper to non-scientific family and friends?

Macrophages are scavenger cells that engulf and ingest dead cells. For the sake of clarity, we would say that there are two main types of macrophages: M1 and M2. M2 macrophages have been shown to promote tumour progression under certain circumstances; whereas, the first ones, M1 macrophages, have been shown to present anti-tumoural effects and, therefore, have been used therapeutically to block cancer development. However, transfer of M1 macrophages has not reached the expected results as many of them become rapidly undetectable in the tumour microenvironment. The reasons behind this problem are poorly understood, and that is exactly what we have tried to elucidate in this study. We have gathered some evidences that suggest that this could be due to the elimination of M1 by M2 macrophages. Particularly, we have observed that under tumoural conditions (culturing cells with tumour-conditioned medium) M1 macrophages undergo apoptosis (a kind of programmed cell death), whereas M2 macrophages remain alive. Interestingly, after that, M2

macrophages eliminate M1 leftovers by engulfment, thus reducing their numbers significantly. These observations have been done *in vitro* (in cell culture) and therefore we cannot conclude that this is the actual cause of M1 elimination *in vivo*, but at least suggest that this might be an important factor that should be taken into account when studying the complex environment of a tumour.

> **"It is amazing to contemplate life as it moves, breath-taking moments of this kind compensate the long-lasting hours that research demands."**

What are the potential implications of these results for your field of research?

The tumour microenvironment is a complex system where pro- and anti-tumoural forces are in competition. Much progress has been made in trying to understand how this relates to immunity. Actually, there are many studies showing the interaction between immune cells and cancer cells, and how this affects tumour progression as a whole. However, the interaction among immune cells and the impact this could have on tumour progression has been largely overlooked. This paper tackles this problem and shows some evidences that, in our opinion, should be considered when planning anti-cancer therapies. Particularly, our results suggest that engulfment might be playing an important role in regulating tumour progression. The elimination of M1 by M2 macrophages might have implications in the way a tumour develops as the balance between pro- and anti-tumoural forces is shifted towards progression. In summary, the interactions between macrophage in the tumour microenvironment should be taken into account for therapies to be well thought out.

What has surprised you the most while conducting your research?

The most surprising thing to me was to be able to see engulfment events. It is amazing to contemplate life as it moves, breath-taking moments of this kind compensate the long-lasting hours that research demands. Another piece of evidence that was intriguing during our studies was the effect of tumour-conditioned medium on the induction of M1-autonomous cell death and M2-driven engulfment. In most cellular biology studies, we focus our attention on cellular behaviour, but I do not normally pay attention to the extent to which cells are influenced by their environment. This has clearly deepened and broadened my understating of the cellular world and its complex interactions with the surroundings.

What, in your opinion, are some of the greatest achievements in your field and how has this influenced your research?

Cell competition was initially discovered in *Drosophila melanogaster* but now evidence shows that the molecular players are conserved in higher eukaryotes as well, including mammals. In the field of oncology, cell competition has help us understand pre-tumoural stages, as one mutation could endow one single cell with a competitive advantage so that it can kill and expand at the expenses of the surrounding cells, populating a given tissue without affecting the total number of cells – a process called field cancerization. Interestingly, the notion of cells struggling for survival resembles that of Darwin's theory of evolution and suggests a correlation between animal and cellular behaviour. This concept is particularly interesting when considering the complex tumour environment, where cells strive and try to endure in the tissue. Based on this previous work we postulated that M1 and M2 macrophages interactions could reproduce some aspects of this cellular behaviour and therefore help us explain why transfer of M1 macrophages has not reach the expected results in oncological treatments.

What changes do you think could improve the professional lives of early-career scientists?

Most laboratories function based on a pyramid-like structure with the boss at the top and the postdocs and pre-doctoral students underneath. I think that the inverse situation would be preferable: bosses helping those below to reach places where he or she cannot reach, taking the best out of everyone. In relation to this, there is the concept of mentoring, which I think would be interesting to implement in laboratory daily life: someone who gives orientation and advice based on experience to help others further develop their skills.

What's next for you?

I have been working as a scientist for more than 10 years now and I still have the same passion for science, so I hope I can continue doing so for many years and continue getting to know the mysteries of life.

Reference

Lolo, F.-N., Rius, C. and Casas-Tintó, S. (2017). Elimination of classically-activated macrophages in tumor-conditioned medium by alternatively-activated macrophages. *Biol. Open* **6**, doi:10.1242/bio.027300.
